# How to Avoid the Detachment of Threads of Varnish during Production, through Cutting and Drawing, in the Manufacture of Lids with a ‘Twist-Off’ Mechanism Used for the Closure of Glass Containers

**DOI:** 10.3390/ma14185434

**Published:** 2021-09-20

**Authors:** Florentino Alvarez-Antolin, Laura Francos-Garrote, Alejandro Gonzalez-Pociño, Alberto Cofiño-Villar

**Affiliations:** Department of Material Science and Metallurgical Engineering, University of Oviedo, Independencia 13, 33004 Oviedo, Spain; laura@itemat.net (L.F.-G.); gonzalezpalejandro@uniovi.es (A.G.-P.); UO229780@uniovi.es (A.C.-V.)

**Keywords:** tinplate, hairing, grammage of varnish, cutting, drawing, “twist off” lids, curing temperature, design of experiments

## Abstract

The lids of glass containers which have a ‘twist-off’ mechanism are manufactured from tinplate through a process of cutting and drawing. Previously, the tinplate was protected with a double layer of a certain epoxy-phenolic varnish. During cutting, the detachment of threads of varnish is produced, and these may reach more than 150 microns in diameter. These threads stick to the equipment, thus hindering the shaping process. After manufacturing thousands of lids, stops must inevitably be made in production in order to clean machinery. Through the application of a fractioned design of experiment (DoE) application, carried out on an industrial scale, the effect of a number of factors on the detachment of threads of varnish was studied. Some to these factors refer to coating, others to the substratum and others to the process of cutting and drawing. It is concluded that the detachment is greater in the disk areas which are parallel to the forward direction of the production line. This problem could be substantially reduced, and even eliminated, if the direction of the rolling of the sheet metal were perpendicular to that of the forward direction of the production line, if the blank-holder is situated at 4 bar, if the time between the curing process and cutting is no more than 3threedays, if the clearance in the cutting is situated at 0.06 mm, and if the grammage of the varnish and the grammage of the layer of tin are increased.

## 1. Introduction

**Figure 1:**
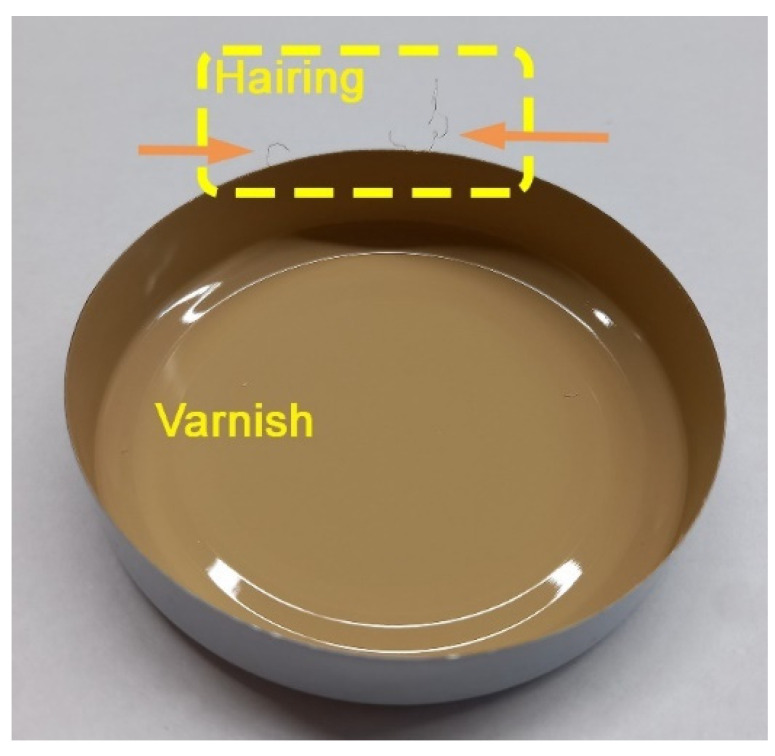
Lid formed after the processes of cutting and drawing.

Glass container lids for use in the food industry are made of tin sheets with a thickness of between 0.14 and 0.18 mm [1]. The production is done with a cutting process and through punching and drawing. The steel presents a wholly ferritic microstructure, with an elastic limit of between 580 and 620 MPa [2]. These are cold rolled, recrystalised, ferritic steels. Before coating with Sn, they undergo a second cold rolling in order to increase the elastic limit. The protective layer of the tinplate is around 3 g/m^2^ on both sides. Tinplate has shown some processing problems because the tin tends to adhere to the cutting and drawing tools, significantly increasing friction between the main components of the tool (punch and die) and the tinplate being formed [3]. Metals are not inert to foodstuffs, so tinplate is coated with protective varnishes to prevent metal-food interaction and migration of metal components [4]. The tinplate is protected with two layers of a certain coating [5,6]. This is a double layer made of epoxy-phenolic varnish which shows a high capacity for adhesion to the substratum [7,8]. The application of this coating is carried out in two stages. In order to do this, the tinplate sheets are put through rollers which apply the coating on both sides of the tinplate. The second layer is dyed with titanium oxide. Curing takes place after the application of each layer. The curing temperature is around 180–200 °C. Subsequently, through a process of cutting and drawing, a ‘lid shape’ is formed [9], see Figure 1. From this lid, by folding, the lug of the ‘twist-off’ caps are forming [10]. This allows air-tight sealing of the glass container.

**Figure 2:**
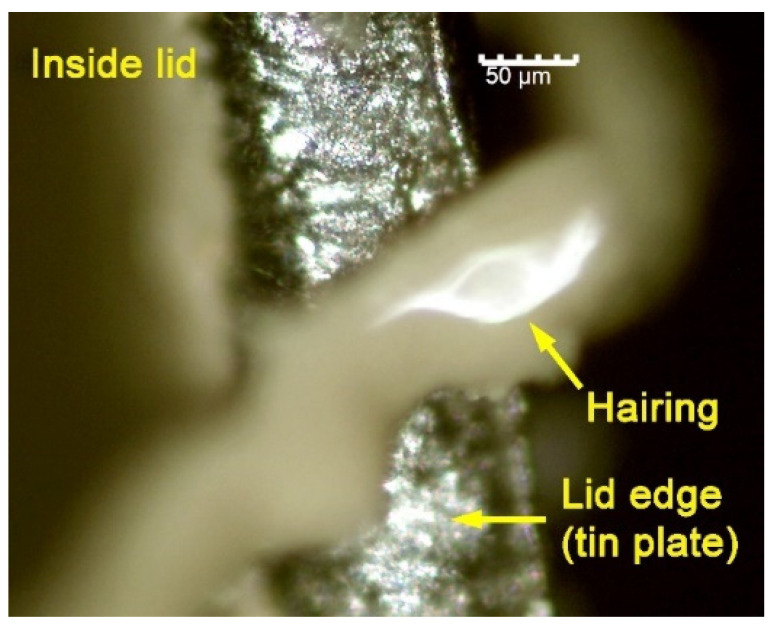
Varnish thread situated on the edge of the lid.

**Figure 3:**
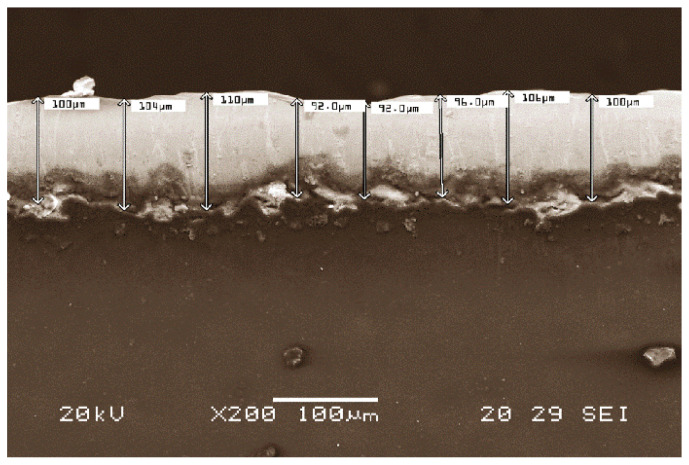
Thickness of the layer which remains without varnish on the edge of the lid after its detachment during the cutting process. It may be observed how this thickness exceeds 100 microns. The image was taken with a scanning electron microscope (SEM).

During cutting with punch (blanking), the detachment of threads of varnish is produced on the circumferential cutting edge of the disk (see Figure 1 and Figure 2) This detachment is a consequence of the deformation suffered by the cutting surface, which is formed by the substratum and the varnish itself. These threads are very fine, with an approximate diameter of some 150–200 microns. The ‘thread’ of unstuck varnish adheres to the machinery, thus slowing down the production process. After having manufactured thousands of lids, it is inevitable to have to stop for maintenance and cleaning of the machinery. This phenomenon of delamination of the varnish is common to industry in this sector and it is known as ‘hairing’. Figure 3 shows an example of the thickness of the ‘layer’ which has remained without varnish on the edge of the lid, due to the detachment of threads of varnish. We define this parameter as ‘thickness of the delaminated layer’.

Despite being a generalised defect in the industry in this sector, at the beginning of this research project the factors which cause ‘hairing’ were unknown. This phenomenon is assumed to be part of the inherent costs of the manufacturing process. An industrial problem was being faced whose causes were unknown and which appears to be outside the more general theoretical concepts related to the microstructure and properties of tinplate or with properties of the varnish which is applied to the tin. ‘Hairing’ could be a consequence of a more complex process of forming than was expected. In this context, it could be more effective to employ an experimental methodology based on deliberate, planned and controlled variation of different factors in the manufacturing process, within a certain range of interest, and on analysis and later interpretation of the obtained results.

During the process of cutting and drawing, there are a number of parameters of manufacturing that could influence ‘hairing’. Some refer to coating, others to the substratum and others to the process of cutting and drawing. In an initial study, it was concluded that ‘hairing’ was not uniform, but rather that it was lower on the part of the cutting disk that coincided with the perpendicular direction forward in the production line. Also, the most influential factor in ‘hairing’ was the pressure of the blank-holder [9]. The influence on “hairing” was also detected, although less significant, of the thickness of the tinplate and the grammage of the varnish. The habitual blank-holder pressure in the manufacturing process was 5 bars and it was shown that a decrease to 3 bars considerably reduced ‘hairing’. The grammage used in the varnish was 12 g/m^2^ and the thickness of the sheet metal was 0.18 mm. It was detected that an increase of 15 g/m^2^ in grammage and a decrease in the thickness of the tinplate to 0.16 mm brought about an additional complement to the effect of the reduction of bank-holder pressure on ‘hairing’. In that first study, the effect of the thickness of the film of Sn on ferritic steel was not analysed.

In this study, as a continuation of that previous study, the aim is to analyse the effect of various parameters of manufacturing and to situate these factors at those levels which allow for a maximum reduction in ‘hairing’, or even its elimination entirely. The research method followed was the application of a fractioned design of experiment (DoE), where eight factors were studied from 16 experiments [11]. The research was carried out on an industrial scale and ‘hairing’ was quantified through the thickness of the delaminated layer, using images obtained with optical microscopy. After considering the initial objective and the available resources, this research methodology allows for the planning of the conditions in which a reduced number of experiments were going to be carried out. These would allow determination of the effect of the studied productive factors on a certain industrial response. In this case, the industrial response would be the thickness of the delaminated layer as a consequence of ‘hairing’.

## 2. Materials and Methods

The application of a design of experiment statistical technique aims to deliberately modify normal working conditions to produce changes in some of the studied responses, in this case, the thickness of the delaminated layer. In industrial processes, normally only a few factors are responsible for the greater part of the variations in response. Complete factorial experiments require a high number of experiments, which grows exponentially with regard to the number of factors to be studied. For example, when k factors are analysed, the number of experiments is 2^k^, where 2 is the number of values or levels which are applied to each factor. Fractional factorial designs allow for the study of a large number of factors with a much lower number of experiments. This implies a loss of information of possible interactions between factors, which, in industrial practice, are not usually very significant.

The effect of a factor is defined as the variation of the function response derived separately from each factor. Similarly, the main effects are defined as those effects on the derived function response of each separate factor. That is to say, the main effect of a certain factor is defined as the change of the function response on varying this factor between its level −1 and its level +1. The interactions between two factors are defined as the variation between the average effect of a factor with the other factor at its level −1, and the average effect of this same factor with the other factor at level +1. Analogically, the interactions among various factors would be defined. The importance of the main effects tends to be greater than the importance of the interactions of two factors, and, at the same time, these are greater than the interactions of three factors, and so on. Yates’ algorithm was applied to calculate main and interaction effects [12].

The resolution of a design indicates the level of confounding which occurs in the estimation of its effects [11]. In general, a resolution design R is that in which no effect of Q factors is confounded with another which contains fewer than R-Q factors. For example, if a design of experiment were considered with eight factors and 16 experiments, its resolution would be IV. That is to say, the main effects are confounded with the interactions of three factors. It may be confirmed that 4 (resolution) = 1 (main effects) + 3 (interactions of three factors).

The experimental response is subject to random variation. This variation follows a normal law where its standard deviation reflects experimental error. The effects are linear combinations of responses, so that, by application of the Central Theorem of Limit, they follow a normal law. It must be taken into account that the linear combination of two normal independent random variables follows a normal law. From this normal law, the distribution function associated with it can be graphically represented. If it is represented on the scale of a ‘normal probability plot’, a straight line would appear [13,14,15,16,17]. If all the effects were not significant, these would follow a law N(0,σ) so that they would appear aligned in the previously mentioned representation (normal probability plot). If an effect were significant, this would follow a law N(µ,σ), so that it would not appear aligned with non-significant effects. Those factors which move away from the straight line, above it to the left, show that their level-1 causes an increase in the response function with respect to its level +1. Those significant factors which move away from the straight line, below it to the right, show that their level +1 causes an increase in response function with respect to their level −1.

Table 1 shows the studied factors and levels and Table 2 the matrix of experiments. In the latter table, the sequence of generators and confounding of the DoE is included. The column ‘generators’ indicates the algorithm applied in the construction of columns E, F, G and H [18]. The column ‘restricted confounding pattern’ shows those secondary order interactions whose effects are confounded. For example, in this case, the interactions AB + CG + DH + EF will be confounded under the same effect. Similarly, this occurs with the rest of the interactions of secondary importance which are shown in this column. The columns for signs E, F, G and H, of the experiment matrix have been constructed as the product of columns BCD, ACD, ABC and ABD respectively.

The following factors were studied:

Factor A refers to the blank-holder pressure during the cutting stage.

Factor B refers to the curing sequence. Level −1 is identified as i + e + e + i. The letter “i” refers to the application of a layer of varnish on the interior side of the lid, and similarly, the letter ‘e’ on the exterior side.

Factor C refers to the space between the punch and the matrix during the cutting process.

Factor D refers to the grammage of the varnish.

Factor E refers to the amount of time between finalisation of the curing treatment and the process of cutting and drawing.

Factor F refers to temperature variation in curing on the interior side. Level -1 corresponds with normal conditions, where curing temperature of the two stages of varnish application are 200 °C for the interior side and 180 °C for the exterior side. At level +1 the curing temperature of the interior side is modified, bringing it to 210 °C.

Factor G refers to the relation between the forward direction of the production line and the direction of ferritic steel rolling. At its level −1, the forward direction coincides with the direction of rolling of the sheet of ferritic steel. At its level +1, the forward direction coincides with the direction perpendicular to the direction of rolling.

Factor H refers to the thickness of the Sn layer, represented by grammage (g/m^2^).

The mathematical model chosen for estimating the response functions was that of ‘interactions of two factors’ This model is formed by the main effects and interactions of two factors. It is defined as follows:

$$\begin{array}{l} Y=B_{0}+B_{1}x_{1}+B_{2}x_{2}+B_{3}x_{3}+B_{4}x_{4}+B_{5}x_{5}+B_{6}x_{6}+B_{7}x_{7}+B_{8}x_{8}\\ +B_{12}x_{1}x_{2}+B_{13}x_{1}x_{3}+B_{14}x_{1}x_{4}+B_{15}x_{1}x_{5}+B_{16}x_{1}x_{6}+B_{17}x_{1}x_{7}+B_{18}x_{1}x_{8}\\ +B_{23}x_{2}x_{3}+B_{24}x_{2}x_{4}+B_{25}x_{2}x_{5}+B_{26}x_{2}x_{6}+B_{27}x_{2}x_{7}+B_{28}x_{2}x_{8}\\ +B_{34}x_{3}x_{4}+B_{35}x_{3}x_{5}+B_{36}x_{3}x_{6}+B_{37}x_{3}x_{7}+B_{38}x_{3}x_{8}\\ +B_{45}x_{4}x_{5}+B_{46}x_{4}x_{6}+B_{47}x_{4}x_{7}+B_{48}x_{4}x_{8}+B_{56}x_{5}x_{6}+B_{57}x_{5}x_{7}+B_{58}x_{5}x_{8}\\ +B_{67}x_{6}x_{7}+B_{68}x_{6}x_{8}+B_{78}x_{7}x_{8} \end{array}$$

From this model, the optimum combination of the levels of each factor could be determined. This is shown in Table 1. This allows maximisation or minimisation of the response function. The software used for this was Statgraphics Centurion XVI, version 16.1.03.

**Figure 4:**
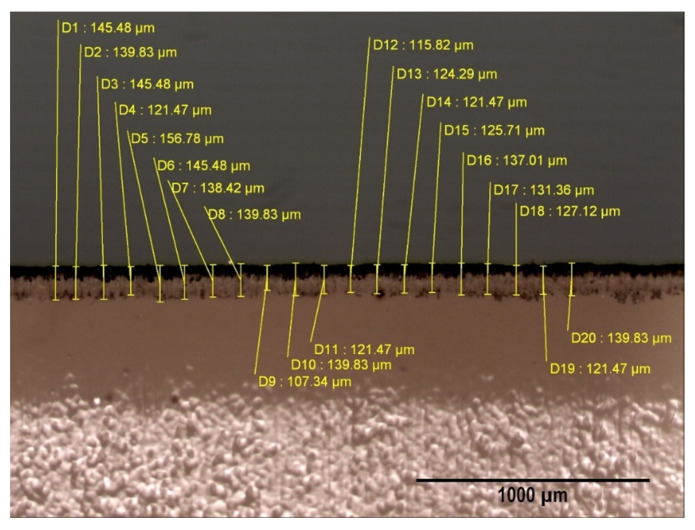
Example of measurement carried out on one of the fragments. In this case it corresponds with experiment 10. This image was obtained using an optical microscope with a magnification of 50.

In each of the 16 experiments, five lids were analyzed. Four fragments set out in perpendicular positions were extracted from each of these along the circumference perimeter of the lid. Two of the fragments were situated in the forward direction of manufacturing and the other two perpendicular to the forward direction. The approximate dimensions of these fragments were 5 mm × 10 mm. The part of the fragment which corresponds with the exterior edge of the lid was observed through an optical microscope. The samples were observed with a magnification of 50. In each of the fragments, 20 measurements were taken, which is equivalent to 80 measurements of each lid and a total of 400 measurements in each experiment. The thickness of the delaminated layer is not uniform around the edges of the fragments, so measurements were taken with a stencil which positioned the 20 measurement points, thus guaranteeing the randomness of the obtained results. The thickness of the delaminated layer was determined as the distance between the edge of the fragment and the point where the lid reaches an apparently uniform density of varnish. Figure 4 shows an example of these measurements.

## 3. Results

**Figure 5:**
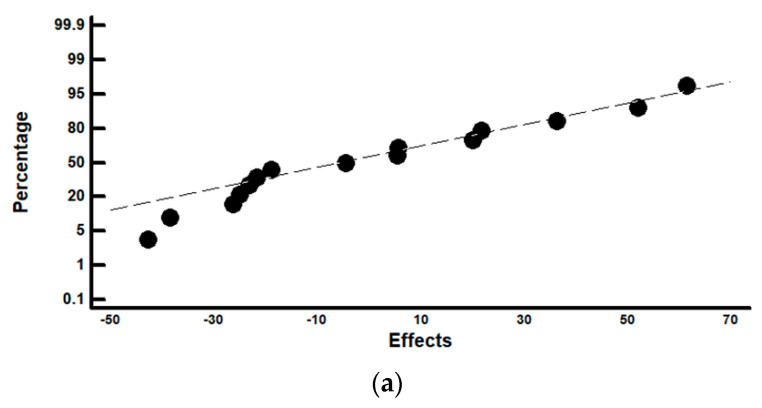

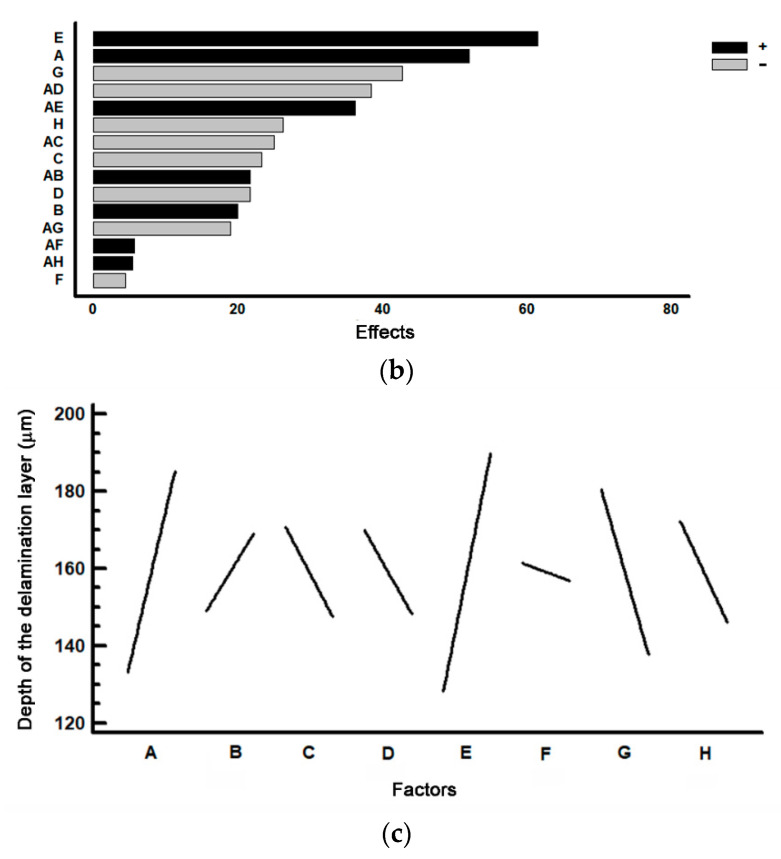
(**a**) Representation of effects on the thickness of the delaminated layer on a normal probability plot; (**b**) Representation of the effects in a Pareto diagram; (**c**) Graph of the main effects.

**Figure 6:**
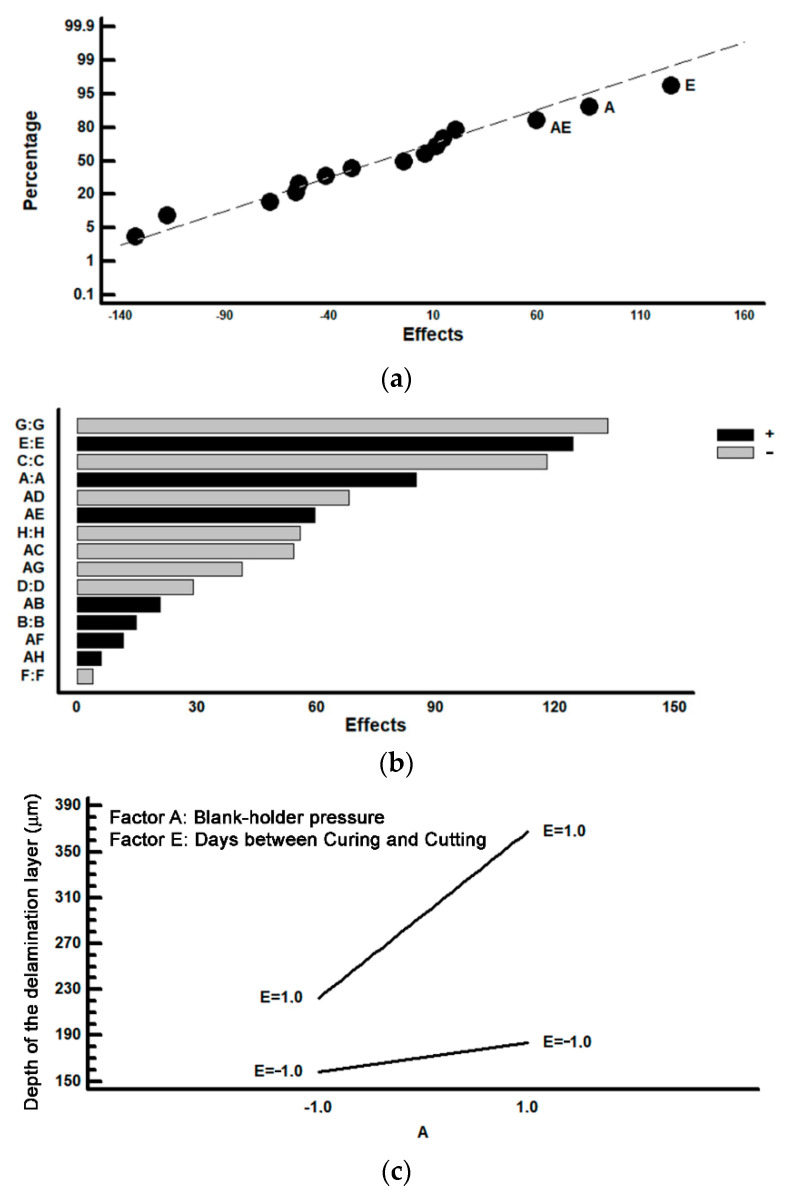
Analysis according to forward direction of the production line; (**a**) Representation of the effects on a normal probability plot; (**b**) Representation of the effects in a Pareto diagram; (**c**) Analysis of the direction AE.

**Figure 7:**
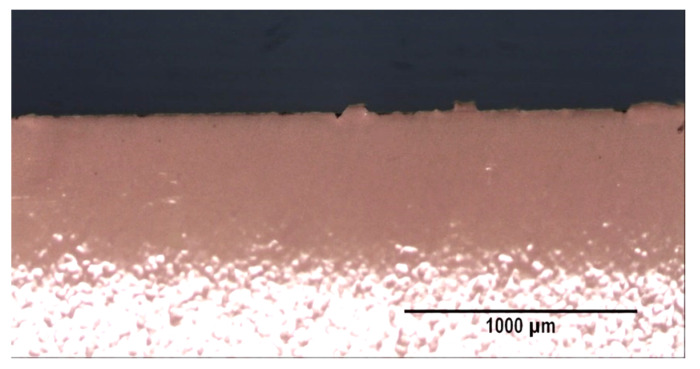
Lack of ‘hairing’ in the lid manufactured with the combination of factors shown in Table 5. Micrograph taken of the area parallel to the production line.

Table 3 shows the results obtained, as a consequence of measuring the average thickness of the delaminated layer (column titled ‘total’). Also, the results are shown as a consequence of considering that thickness in accordance with the pieces situated in a direction parallel to that of the rolling or in a perpendicular direction. Figure 5, Figure 6 and Figure 7 show a graphic interpretation of these results. The first conclusion to be made with respect to these results is that the thickness of the delaminated layer is always greater in the parallel forward direction of the production line.

Figure 5 shows the results corresponding to the analysis of the total perimeter of the lids. In Figure 5a the effects are represented according to a normal probability plot. Figure 5b represents the effects by means of a Pareto diagram. Figure 5c shows the range of the main effects. It may be observed that none of the analysed factors has a significant effect. However, as a consequence of applying the mathematical model of the interactions of two factors, and by analysing all the possible combinations of the levels, the optimum combination which allows for minimising the delaminated layer may be determined. Table 4 shows these results.

Implicitly, these results include two different behaviors. On the one hand, the “delamination” suffered according to the direction parallel to the manufacturing line and, on the other hand, according to the direction perpendicular to the manufacturing line.

Figure 6 shows the results corresponding to the analysis of the section of the perimeter of the lids which is found in the direction parallel to the direction of rolling. In Figure 6a the effects are represented in a normal probability plot. Figure 6b represents the effects in a Pareto diagram. Figure 6c shows an analysis of the interaction AE, which demonstrates a significant effect on the thickness of the delaminated layer. In Figure 6a it may be seen that a significant effect is to be seen in factors E (time between the curing stage and the cutting and drawing stage), A (blank-holder pressure) and the interaction between AE. It is concluded that when both factors are situated at their levels +1 is when a greater thickness in the delaminated layer is produced, that is to say, greater ‘hairing’. Figure 6c gives evidence that if both factors are situated simultaneously at their +1 levels, the loss of varnish increases. As a consequence of the application of the mathematical model of interactions of two factors, and, by analysing all the possible combinations of the levels, the optimal combination may be determined. This would allow the elimination of ‘hairing’, in accordance with the parallel forward direction of the production line. Table 5 shows the optimal combination. It can be seen that Factor A is significant and increases the thickness of the delaminated layer when it is situated at its level +1 (4 bar). Nevertheless, on considering the combination with the rest of the factors, it is recommendable to maintain Factor A at its +1 level, as long as Factor E is situated at its −1 level. Figure 7 shows an example of the lack of ‘hairing’ on following the combination of levels as specified in Table 5.

**Figure 8:**
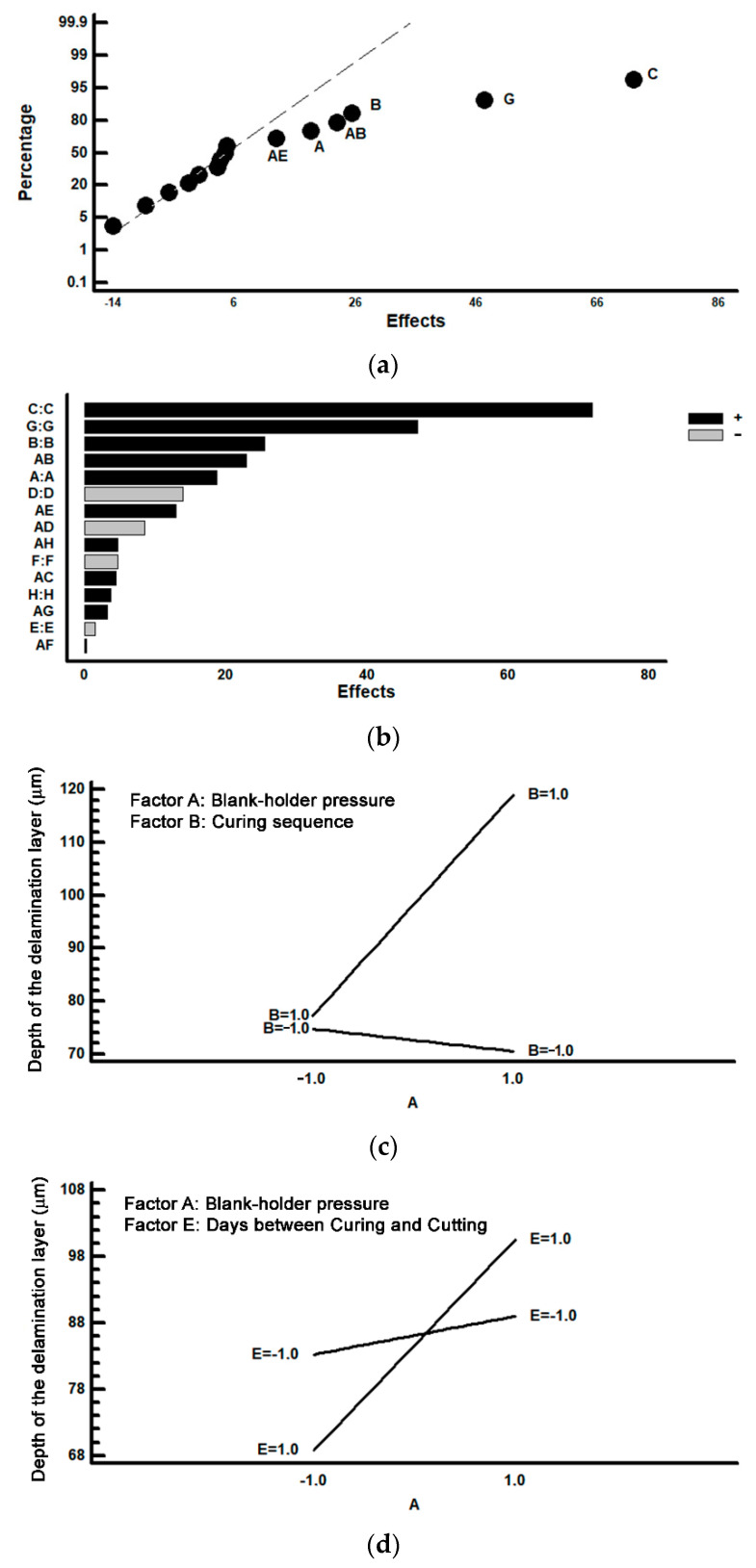
Analysis according to the perpendicular forward direction of the production line; (**a**) Representation of the effects on a normal probability plot; (**b**) Representation of the effects in a Pareto diagram; (**c**) Analysis of the interaction AB; (**d**) Analysis of the interaction AE.

Figure 8 shows the results corresponding to the analysis of the section of the perimeter of the lids which is found in a perpendicular direction to the forward direction of manufacture. In Figure 8a the effects are represented on a normal probability plot. In Figure 8b the effects are represented in a Pareto diagram. Figure 8c shows the analysis of the interaction AB and Figure 8d shows the analysis of the interaction AE. Both interactions show evidence of a significant effect on the thickness of the delaminated layer. In Figure 8a it can be observed that various factors have a significant effect: C (width in the cutting process), G (the relation between the forward direction of the production line and the direction of rolling), B (curing sequence), A (blank-holder pressure) and interactions AB and AE. It is concluded that when all these factors are situated at their +1 level is when the greater thickness of the laminated layer is produced. The analysis of interactions AB and AE are shown respectively in Figure 8c,d.

As a consequence of applying the mathematical model to the interactions of two factors, and of analysing all possible combinations at all levels, the optimal combination for minimising the thickness of the delaminated layer can be determined. Table 4 shows the optimal combination of levels which would help to avoid loss of varnish. It can be seen that when factors A and B are simultaneously situated at their +1 levels, a complementary delamination is produced. Similarly, it is observed that, if factors A and E are situated simultaneously at their +1 levels, an additional increase in the thickness of the delaminated layer is also produced. At the same time, the most significant effect of factors C (space between the cutting matrix and the punch) as well as G (the relation between the direction of rolling and the area of the cutting disk) must also be highlighted. In this way, if the space is wide (0.06 mm), greater delamination is produced. On the other hand, in the areas of the cutting disk perpendicular to the direction of lamination, a significant increase in the thickness of the delaminated layer is also produced. Table 6 shows the optimal combination of levels which lead to the elimination of loss of varnish.

As has been previously analysed, the phenomenon of ‘hairing’ is more marked in the lid depending on the forward direction of the manufacturing line. Depending if the direction of analysis is parallel or perpendicular to the direction of advance of the manufacturing line, it may be deduced that less ‘hairing’ occurs in the direction perpendicular to the direction of rolling. Thus, it may be concluded that the phenomenon of ‘hairing’ will be reduced if the direction of rolling of the sheet metal is situated in a perpendicular direction to the forward direction of the production line. This is reflected in Table 4

## 4. Discussion

**Figure 9:**
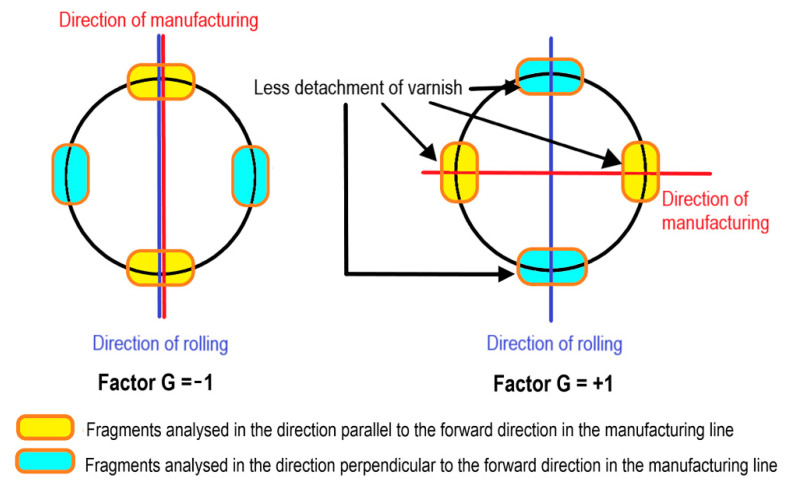
Results after separate analysis of the fragments parallel to or perpendicular to the forward movement of the production line. The loss of varnish is much lower when the direction of lamination and the forward movement of the production line are perpendicular.

The ‘hairing’ phenomenon is a consequence of a more complex process than that which had been expected. At the same time, in the analysis of this phenomenon there were no previous references which allowed for narrowing down the factors of analysis which intervene in the manufacturing process. All of this justifies the use of a research methodology based on design of experiment. During the cutting process with a punch (blanking), the blank-holder should act as a clamp. If the pressure is high, then excessive deformation at the edge of the disk after cutting could be produced, which may affect adherence between the varnish and the substrate. If pressure were low, the blank-holder would act as a guide, thus allowing movement of the lid during cutting. In previous research, two levels of pressure were analysed: 3 and 6 bars [9]. In that case, the 3 bar pressure was more effective compared to 6 bar pressure. In this research, pressure of the blank-holder has been optimised at 4 bars. The clearance between the blades is an important variable in the cutting operation. If this were excessive, the surface of the metal would suffer high plastic deformation before fracturing. If this were small, the surface of fracture would be irregular and would require greater cutting energy. As a result of statistical analysis, it appears that lower ‘hairing’ would be achieved with a clearance of 0.06 mm. As a consequence of results shown in Table 3, it may be concluded that the phenomenon of ‘hairing’ is more pronounced in the area of the lid which is situated in the same direction as the forward movement of the production line. However, this effect could be minimised, or even eliminated, if the tin lid is situated in such a way that the direction of lamination is perpendicular to the forward movement of the production line. In effect, in Figure 9 it can be observed that, when considering exclusively the fragments situated in a parallel direction to the forward movement of the production line, a lower loss of varnish is produced in the direction perpendicular to that of lamination. Similarly, if the fragments situated in the direction perpendicular to the forward movement of the production line are considered exclusively, a lower loss of varnish occurs in the direction parallel to that of lamination. This confirms the result shown in Table 4, where it is shown that the loss of varnish is lower when the direction of the forward movement of the production line is perpendicular to that of lamination. This behaviour is difficult to explain. It could be a consequence of residual forces derived from the cold rolling process of the tinplate sheet and its relation with some ‘defect’ related to the forward movement of the production line. For example, there could be a slight fault in parallelism between the surface of the punch or the blank-holder and the surface of the tin sheet during forward movement. Perhaps this is due to the movement of the tinplate sheet itself during this forward movement. These residual forces are found in equilibrium. During the cutting (blanking) process, this equilibrium may be altered and may generate a more marked elastic reaction in the direction of lamination when this is parallel to the forward movement of the production line.

The substrate to which the varnish adheres is the layer of Sn. It is shown that the grammage of the varnish has great influence, favouring adherence between the coating and the substrate during cutting (blanking). Similarly, it would be favourable to increase the thickness of the layer of Sn, characterised by its grammage (g/m^2^), thus favouring adherence between the coating and the substrate during the deformations which take place during cutting (blanking).

## 5. Conclusions

The tinplate lids with a “twist off” mechanism for the closing of glass containers are manufactured from sheet metal through a process of cutting, by punching, and drawing. Previous to this, the tinplate is protected with a double layer of epoxy-phenolic varnish. During cutting, the phenomenon of ‘hairing’ occurs. This refers to the detachment of threads of varnish from the edge of the disk. These may reach 150 microns in diameter, and they cause periodic maintenance stops in order to clean machinery. Through a fractioned design of experiment, the effect of several parameters of manufacturing relating to this phenomenon were studied. These factors were situated at those levels which allowed maximum reduction, and even the elimination of ‘hairing’. The main conclusions are:

1. It has been shown that ‘hairing’ is more marked in the areas of the disk which are parallel to the forward direction of the production line, rather than in their perpendicular areas. The ‘hairing which is produced in these areas can be reduced if:
  - The rolling direction of the sheet is perpendicular to the advance of the manufacturing line.
  - If the blank-holder pressure is situated at 4 bar.
  - If the time between the curing process and cutting is no longer than three days.
2. It has been shown that the order of the curing stages has an influence on ‘hairing’.
3. The elimination of ‘hairing’ could be achieved in the disk area perpendicular to the forward direction of the production line. For this, the following is additionally recommended:
  - A space between the punch and the cutting matrix of 0.06 mm
  - 20 g/m^2^ of grammage in the varnish
  - And a high grammage of the Sn layer. Around 11.2 g/m^2^.

## Figures and Tables

**Table 1:** Factors and Levels.

Factors		Levels	
Manufacturing Parameters		−1	+1
A	Blank-holder Pressure (bar)	3	4
B	Curing Sequence	i + e + e + i	i + i + e + e
C	Clearance of cut (mm)	0.03	0.06
D	Grammage of Varnish (g/m^2^)	17	20
E	Time between Curing and Cutting (days)	3	6
F	Temperature of curing of the interior side (°C)	200	210
G	Direction of rolling of the tinplate sheet	Parallel to the Manufacturing line	Perpendicular to the Manufacturing line
H	Grammage of the layer of Sn (g/m^2^)	2.8	11.2

**Table 2:** Experimental Matrix.

Experiment	A	B	C	D	E	F	G	H	Generators	Confounding Patterns
1	−1	−1	−1	−1	−1	−1	−1	−1	E = BCD F = ACD G = ABC H = ABD	A B C D E F G H AB + CG + DH + EF AC + BG + DF + EH AD + BH + CF + EG AE + BF + CH + DG AF + BE + CD + GH AG + BC + DE + FH AH + BD + CE + FG
2	1	−1	−1	−1	−1	1	1	1		
3	−1	1	−1	−1	1	−1	1	1		
4	1	1	−1	−1	1	1	−1	−1		
5	−1	−1	1	−1	1	1	1	−1		
6	1	−1	1	−1	1	−1	−1	1		
7	−1	1	1	−1	−1	1	−1	1		
8	1	1	1	−1	−1	−1	1	−1		
9	−1	−1	−1	1	1	1	−1	1		
10	1	−1	−1	1	1	−1	1	−1		
11	−1	1	−1	1	−1	1	1	−1		
12	1	1	−1	1	−1	−1	−1	1		
13	−1	−1	1	1	−1	−1	1	1		
14	1	−1	1	1	−1	1	−1	−1		
15	−1	1	1	1	1	−1	−1	−1		
16	1	1	1	1	1	1	1	1		

**Table 3:** The thickness of the delaminated layer in the total perimeter of the lid, according to the parallel forward direction of the production line. All of the values are in microns.

No.	Total	Effects	Parallel	Effects	Perpendicular	Effects	Effects
1	145	159.12	258	233.0	32	85.37	Average
2	129	5.0	192	85.0	65	18.7	A
3	113	20.0	163	14.7	63	25.5	B
4	351	23.2	636	−117.7	66	72.0	C
5	138	21.7	152	−29.2	124	−14.0	D
6	239	61.5	371	124.2	107	−1.5	E
7	103	−4.5	111	−4.0	96	−4.75	F
8	142	−42.7	98	−133.0	186	47.2	G
9	145	−26.2	284	−56.0	6	3.75	H
10	186	21.7	321	20.7	50	23.0	AB
11	126	−25.0	184	−54.2	68	4.5	AC
12	171	−38.5	297	−68.2	45	−8.5	AD
13	108	36.2	80	59.7	137	13.0	AE
14	103	5.7	147	11.5	60	0.25	AF
15	187	−19.0	292	−41.5	82	3.25	AG
16	160	5.5	142	6.0	179	4.75	AH

**Table 4:** Optimal combination of levels to minimise the thickness of the delaminated layer on the total perimeter of the lid after the cutting and drawing process = 19.25 microns.

Factors		Optimal Level	
A	Blank-holder Pressure (bar)	1	4
B	Curing Sequence	−1	i + e + e + i
C	Clearance of cut (mm)	1	0.06
D	Grammage of Varnish (g/m^2^)	1	20
E	Time between Curing and Cutting (days)	−1	3
F	Temperature of curing of the interior side (°C)	−1	200
G	Direction of rolling of the tinplate sheet	1	perpendicular
H	Grammage of the layer of Sn (g/m^2^)	1	11.2

**Table 5:** Optimal combination of levels for minimising the thickness of the delaminated layer in the direction parallel to the forward direction in the production line, after the cutting and drawing process. In this combination of levels ‘hairing’ would be eliminated in the direction parallel to the production line.

Factors		Optimal Level	
A	Blank-holder Pressure (bar)	1	4
B	Curing Sequence	−1	i + e + e + i
C	Clearance of cut (mm)	1	0.06
D	Grammage of Varnish (g/m^2^)	1	20
E	Time between Curing and Cutting (days)	−1	3
F	Temperature of curing of the interior side (°C)	−1	200
G	Direction of rolling of the tinplate sheet	1	perpendicular
H	Grammage of the layer of Sn (g/m^2^)	1	11.2

**Table 6:** Optimal combination of levels to avoid delamination in the perpendicular forward direction of manufacturing, after the process of cutting and drawing. In this combination, the levels would eliminate the ‘hairing’ in the direction perpendicular to the production line.

Factors		Optimal Level	
A	Blank-holder Pressure (bar)	1	4
B	Curing Sequence	−1	i + e + e + i
C	Clearance of cut (mm)	−1	0.03
D	Grammage of Varnish (g/m^2^)	1	20
E	Time between Curing and Cutting (days)	−1	3
F	Temperature of curing of the interior side (°C)	1	210
G	Direction of rolling of the tinplate sheet	−1	parallel
H	Grammage of the layer of Sn (g/m^2^)	−1	2.8

## Data Availability

Data are contained within the article.
